# Corticosteroid Therapy and Long-Term Outcomes of Post-Infectious Inflammatory Syndrome in Non-HIV Immunosuppressed Cryptococcal Meningitis: A Multicenter Case Series

**DOI:** 10.1093/infdis/jiaf620

**Published:** 2026-01-05

**Authors:** Yuya Ito, Tracey-Ann Hoeltermann, Seher Anjum, Londyn Robinson, Jessica S Little, Michael Kiritsy, Julie M Steinbrink, Andrea Finocchi, Lorne W Walker, Robin K Avery, Shmuel Shoham, Omer E Beaird, Song C Ong, Cornelius N Van Dam, Ina Stephens, Ambar Haleem, Peter R Williamson

**Affiliations:** Laboratory of Clinical Immunology and Microbiology (LCIM), Division of Intramural Research (DIR), National Institute of Allergy and Infectious Diseases (NIAID), National Institutes of Health (NIH), Bethesda, Maryland, USA; Laboratory of Clinical Immunology and Microbiology (LCIM), Division of Intramural Research (DIR), National Institute of Allergy and Infectious Diseases (NIAID), National Institutes of Health (NIH), Bethesda, Maryland, USA; Laboratory of Clinical Immunology and Microbiology (LCIM), Division of Intramural Research (DIR), National Institute of Allergy and Infectious Diseases (NIAID), National Institutes of Health (NIH), Bethesda, Maryland, USA; Department of Medicine, Division of Rheumatology, University of Washington School of Medicine, Seattle, Washington, USA; Harvard Medical School, Dana-Farber Cancer Institute, Boston, Massachusetts, USA; Division of Infectious Diseases, Harvard Medical School, Brigham and Women's Hospital, Boston, Massachusetts, USA; Department of Medicine, Division of Infectious Diseases, Duke University School of Medicine, Durham, North Carolina, USA; Department of Medicine, Division of Infectious Diseases, Duke University School of Medicine, Durham, North Carolina, USA; Academic Department of Pediatrics, Bambino Gesù Children's Hospital, Unit of Immunoinfectivology, IRCCS, Rome, Italy; Department of Systems Medicine, University of Rome Tor Vergata, Rome, Italy; Division of Pediatric Infectious Diseases, Oregon Health and Science University, Portland, Oregon, USA; Department of Medicine, Division of Infectious Diseases, Johns Hopkins University, Baltimore, Maryland, USA; Department of Medicine, Division of Infectious Diseases, Johns Hopkins University, Baltimore, Maryland, USA; Department of Medicine, Division of Infectious Diseases, David Geffen School of Medicine at UCLA, Los Angeles, California, USA; Department of Medicine, Division of Nephrology, University of Alabama at Birmingham, Birmingham, Alabama, USA; Regional Center for Infectious Diseases, Cone Health, Greensboro, North Carolina, USA; Department of Infectious Diseases, University of Virginia Children's Hospital, Charlottesville, Virginia, USA; Department of Medicine, Division of Infectious Disease, University of Wisconsin School of Medicine and Public Health, Madison, Wisconsin, USA; Laboratory of Clinical Immunology and Microbiology (LCIM), Division of Intramural Research (DIR), National Institute of Allergy and Infectious Diseases (NIAID), National Institutes of Health (NIH), Bethesda, Maryland, USA

**Keywords:** *Cryptococcus*, meningoencephalitis, immunosuppressed patients, post-infectious inflammatory response syndrome (PIIRS), corticosteroids

## Abstract

**Background:**

Post-infectious inflammatory response syndrome (PIIRS) is recognized as a cause of neurologic deterioration in previously healthy patients with cryptococcal meningoencephalitis (CM). However, data on non-human immunodeficiency virus (HIV), immunosuppressed patients remain limited.

**Methods:**

Between July 2018 and April 2025, 13 non-HIV immunosuppressed patients with CM who subsequently developed PIIRS were included. Clinical features, Karnofsky performance scores, cerebrospinal fluid (CSF) parameters, and magnetic resonance imaging (MRI) findings were compared at PIIRS diagnosis and during follow-up after corticosteroid therapy.

**Results:**

All patients showed evidence of CNS inflammation, including abnormal CSF, MRI findings, and neurological symptoms such as altered mental status or visual/hearing loss. Corticosteroid therapy was associated with significant improvements in Karnofsky scores at 1 month (*P* = .001), with sustained benefit at 6 and twelve months (*P* = .002); all 10 surviving patients demonstrated resolution of neurological symptoms. CSF inflammatory parameters including white blood cell (WBC) count, protein, and CSF/serum glucose ratio also significantly improved at 1 month. Brain MRI findings also showed a trend toward improvement. All patients remained culture-negative post-PIIRS diagnosis. Three patients died: 1 from complications of alcoholic cirrhosis, the second from liver failure in the setting of systemic lupus erythematosus and immunosuppression, and the third from sepsis after initiation of corticosteroids.

**Conclusions:**

Corticosteroids were associated with improvement in neurological status and neuroinflammation in non-HIV, immunosuppressed patients with PIIRS following CM. These findings support its potential role as salvage therapy in this population and highlight the need for systematic data collection or randomized trials to better guide corticosteroid use.

## Teaser

Corticosteroid therapy improved neurologic function and reduced neuroinflammation in non-HIV immunosuppressed patients with cryptococcal PIIRS. These findings support its potential as salvage therapy and highlight the need for systematic data collection to inform a future clinical trial.

Cryptococcal disease has become the leading cause of nonviral meningitis in the United States due to combination of increased immunosuppression and the control of bacterial disease by vaccines [1–4]. While the incidence in human immunodeficiency virus (HIV)-infected individuals has declined due to prevention and treatment strategies in developed countries, the overall incidence in HIV-negative infections has increased, in part due to increasing numbers of transplant patients as well as other patients receiving immunosuppressive therapy, such as cancer chemotherapy or for autoimmune diseases [4]. Despite effective fungicidal therapy with amphotericin B and flucytosine as well as follow-up consolidation therapy with fluconazole, estimated mortality remains at 30–50% [5]. Those who survive are often left with cognitive, visual and hearing deficits.

Historically, treatment strategies for infectious diseases have prioritized enhancing pathogen clearance. However, increasing attention has been given to the role of host-mediated immune injury, conceptualized as a parabolic balance between effective microbial control and detrimental inflammation [6]. This balance is especially critical in infections of the central nervous system (CNS), where the inflexible confines of the skull limit the brain's capacity to tolerate swelling and inflammation. In both bacterial and fungal infections of the CNS, immune-driven pathology has been well documented [7], particularly in the context of cryptococcal infections [8].

In patients with CM, a clinical syndrome known as a post-infectious inflammatory response syndrome (PIIRS) may emerge after the pathogen has been cleared, most often following initiation of antifungal therapy. This syndrome shares similarities with HIV-associated immune reconstitution inflammatory syndrome (IRIS), which typically follows the introduction of combination antiretroviral therapy [9, 10]. Unlike HIV-IRIS, which arises from restoration of immune function, PIIRS appears to result from ongoing immune activation in response to increased fungal antigens released after therapy or a reduction in immunosuppressive factors such as capsular components [11]. Solid organ transplant recipients also may develop a similar inflammatory syndrome after anti-fungal treatment and may have elements of PIIRS and IRIS, the latter due to alterations in immunotherapy after fungal diagnosis [12].

Previously, we had reported successful treatment of PIIRS with corticosteroids in a single-center cohort of non-immunosuppressed patients; however, the clinical course and management of PIIRS in immunosuppressed individuals, including solid organ transplant recipients is not well described [13]. To address this knowledge gap, we investigated the outcomes of corticosteroid therapy in 13 immunosuppressed, patients with non-HIV who showed poor clinical responses despite standard-of-care antifungal therapy and developed PIIRS.

## METHODS

### Study Design and Participants

This was a multi-center, prospective, observational case series involving 13 consecutive patients from 12 different institutions (Supplementary Table 1) whose physicians contacted the NIH for clinical advice between July 2018 and April 2025 due to lack of significant improvement in clinical condition despite standard-of-care fungicidal therapy. Three have been reported as case reports [14–16]. This case series included HIV-negative, immunocompromised patients who were subsequently treated with corticosteroids after a diagnosis of PIIRS was made. The diagnosis of CM was based on a positive culture for *Cryptococcus* species from cerebrospinal fluid (CSF). PIIRS was defined as the occurrence of 1 or more of the following symptoms after initial therapy with amphotericin B-containing regimens: decline or failure to improve mental or performance status, visual deficits or changes in hearing after or coincident with CSF fungal culture conversion [13]. Patients who had been previously healthy prior to the onset of CM or who were HIV-positive were excluded. Patients were prospectively followed. Data was retrospectively acquired and analyzed by the treating physician and the referring center. The choice between pulse and non-pulse corticosteroid regimens for PIIRS was determined based on the patient's general condition and the risk of infection associated with immunosuppression, in consultation with infectious disease specialists at our institution and collaborating centers.

### Data Acquisition

The following data were collected from each participating institution: age, sex, race, comorbidities, and use of corticosteroids and immunosuppressants. The use of corticosteroids and immunosuppressants was defined according to the criteria established by the European Organization for Research and Treatment of Cancer and the Mycoses Study Group Education and Research Consortium (EORTC/MSGERC) [17]. Clinical data were collected at the time of CM diagnosis, at the time of PIIRS diagnosis, 1 month, 6 months and 1 year after initiation of corticosteroid therapy and included symptoms, laboratory data, CSF parameters, and brain magnetic resonance imaging (MRI) findings. The brain MRI score was calculated by assigning 1 point each for the presence of leptomeningeal enhancement, choroiditis, ependymitis, parenchymal lesions, and hydrocephalus observed on post contrast T1 and FLAIR images. Additionally, the following information was collected: the duration from the onset of CM symptoms to CM diagnosis, the duration from CM diagnosis to PIIRS diagnosis, the duration of corticosteroid treatment for PIIRS, the type and dosage of corticosteroids used for PIIRS.

### Statistical Analysis

Categorical variables were expressed as frequencies and percentages, while continuous variables were expressed as medians with interquartile ranges (IQRs). Wilcoxon matched pairs signed rank tests were conducted to analyze patient parameters. All statistical analyses were conducted using Prism software package (version 10.0; GraphPad Software, Inc., CA, USA). *A P*-value of < .05 was considered statistically significant.

### Patient Consent Statement

All subjects provided informed consent in accordance with institutional policies. According to the guidelines of the National Institutes of Health (NIH), ethical review board approval was not required for retrospective case series in which patient data were de-identified and collected as part of routine clinical care [18].

## RESULT

### Demographic Data

A total of 13 patients were included in the analysis. Demographic data are shown in Table 1. The median age of the cohort was 52 years (IQR, 35–60 years), and 9 patients (69.2%) were male. Patients were all significantly immunosuppressed with 1 patient having a genetic mutation (CD40 ligand), 8 patients (61.5%) having a history of solid organ transplantation, 2 patients (15.4%) with hematologic malignancies, and 2 patients (15.4%) having connective tissue diseases on immunosuppressants (Table 1). Twelve patients (92.3%) had positive CSF cultures of *Cryptococcus neoformans* at the time of CM diagnosis with the remainder diagnosed by a cryptococcal lateral flow assay (CrAg LFA, Immuno-Mycologics, inc., Norman, OK, USA). The median duration from CM symptom onset to CM diagnosis was 19 days (IQR, 8–44 days), and the median duration from CM diagnosis to PIIRS diagnosis was 17 days (IQR, 9–100 days) (Table 1).

**Table 1.** Demographic Data of Patients with Post-infectious Inflammatory Response Syndrome (PIIRS)

Variables	N = 13
Age, median (IQR)	52 (35–60)
Male, n (%)	9 (69.2)
Race, n (%)	…
Caucasian	7 (53.8)
African	1 (7.7)
Hispanic	3 (23.1)
Asian	2 (15.4)
Comorbidities, n (%)	…
Post transplantation	8 (61.5)
Hematologic malignancy	2 (15.4)
Solid organ malignancy	1 (7.7)
Connective tissue diseases	2 (15.4)
Chronic kidney disease	5 (38.5)
Diabetes mellitus	5 (38.5)
Chronic liver diseases	3 (23.1)
Hyper IgM syndrome^a^	1 (7.7)
Species, n (%)	…
*C. neoformans*	12 (92.3)
*Cryptococcus gattii*	1 (7.7)
Antifungals	…
AmphotericinB + flucytosine	5 (38.5)
Fluconazole	8 (61.5)
Median time from CM symptom onset to CM diagnosis in days (IQR)	19 (8–44)
Median time from CM diagnosis to PIIRS diagnosis in days (IQR)	17 (9–100)

Abbreviations: IQR, interquartile range; IgM, immunoglobulin M; CM, cryptococcal meningoencephalitis; PIIRS, post-infectious inflammatory response syndrome. ^a^Hyper IgM syndrome is a primary immunodeficiency caused by defective class-switch recombination due to mutations in the CD40 ligand (CD40L) gene.

### Clinical Characteristics of Patients at the Time of Post-Infectious Inflammatory Response Syndrome Diagnosis

Six patients had altered mental status evidenced by abnormal Glasgow Coma Scale and all had reduced Karnofsky scores (median score of 40) at the time of PIIRS diagnosis (Table 2). In addition, 9 patients (69.2%) presented with headache, 8 patients (61.5%) reported nausea, 4 patients (30.8%) experienced a change in hearing and 3 had visual changes (23.1%) (Table 2). Two patients had only visual symptoms, and none had hearing issues exclusively in the absence of mental status changes. Three had intractable headaches and nausea/vomiting as their only symptom complex. CSF parameters were abnormal in all patients with elevated opening pressure evident in 5 of 11 measured, elevated white blood cell (WBC) count in 11 with a bloody sample in 1, elevated protein levels in 11, with the CSF/serum glucose ratios decreased. Contrast brain MRIs were obtained and were abnormal in all cases with leptomeningeal enhancement evident in 7 patients (53.8%), parenchymal lesions in 5 patients (38.5%), and hydrocephalus in 4 patients (30.8%) (Table 2). At the time of PIIRS diagnosis, immunosuppressive agents had already either been discontinued or reduced, with a median prednisone dose of 0.09 mg/kg/day compared to 0.14 mg/kg/day at the time of CM diagnosis. Mycophenolate mofetil was discontinued in most patients on this agent in order to decrease overall immunosuppression [19].

**Table 2.** Clinical Characteristics of Patients at the Time of Cryptococcal Meningoencephalitis (CM) Diagnosis and at Post-infectious Inflammatory Response Syndrome (PIIRS) Diagnosis

Variables	At CM Diagnosis (N = 13)	At PIIRS Diagnosis (N = 13)
Glasgow coma scale, median (IQR)	15 (13–15)	15 (12–15)
Karnofsky performance scales^a^, median (IQR)	−	40 (20–50)
Symptoms, n (%)	…	…
Altered mental status	8 (61.5)	6 (46.2)
Vision deficit	5 (38.5)	3 (23.1)
Change in hearing	2 (15.4)	4 (30.8)
Fever	7 (53.8)	3 (23.1)
Headache	12 (92.3)	9 (69.2)
Nausea/Vomiting	11 (84.6)	8 (61.5)
CSF parameters, median (IQR)	…	…
Opening pressure (cmH_2_O)	32 (20–42)^d^	19 (14–32)^d^
WBC (/mm^3^)	83 (40–150)	71 (30–236)
(/mm^3^), (n = 11)	22.8 (3.60–67.5)	2.86 (1.40–48.3)
Lymphocytes (/mm^3^), (n = 11)	24.6 (7.47–75.7)	24.9 (14.4–141.6)
CSF-Serum glucose ratio (CSF/Serum)	0.30 (0.20–0.46)	0.33 (0.17–0.46)
Protein (mg/dL)	113 (65–161)	188 (78–400)
Abnormal brain MRI findings^a^, n (%)	…	…
enhancement	−	7 (53.8)
Choroiditis	−	1 (7.7)
Ependymitis	−	1 (7.7)
Parenchymal lesions	−	5 (38.5)
Hydrocephalus	−	4 (30.8)
Other^b^	−	7 (53.8)
Immunosuppressants, n (%) and doses, median (IQR)	…	…
Corticosteroids	7 (53.8)	3 (23.1)
Dosage (mg/kg/d)^c^	0.14 (0.08–0.36)	0.09 (0.08–0.73)
Tacrolimus	7 (53.8)	5 (38.5)
Dosage (mg/kg/d)	0.08 (0.02–0.18)	0.03 (0.01–0.06)^e^
Cyclosporine	0 (0)	1 (7.7)
Mycophenolate Mofetil	7 (53.8)	1 (7.7)
Dosage (mg/d)	1080 (1000–1500)	1500^f^
Cyclophosphamide	1 (7.7)	0 (0)
Rituximab	1 (7.7)	0 (0)
Chemotherapy	2 (15.4)	0 (0)

The normal Glasgow Coma Scale score is 15 (range 3–15); the normal Karnofsky Performance Scale score is 100 (range 0–100).

Abbreviations: CM, cryptococcal meningoencephalitis; CSF, cerebrospinal fluid; Hb, hemoglobin; IQR, interquartile range; MRI, magnetic resonance imaging; PIIRS, post-infectious inflammatory response syndrome; WBC, white blood cell.

^a^Karnofsky performance scale and brain MRI findings were available only at the time of PIIRS diagnosis.

^b^Infarction, edema, and vessel enhancement.

^c^All corticosteroid dosages, except for pulse corticosteroid therapy, were expressed as prednisone equivalents.

^d^Results were available for 11 patients.

^e^Results were available for 4 patients.

^f^Only 1 patient received a dose of 1500 mg; value shown as raw data.

Compared with the time of CM diagnosis, fewer patients presented with fever, headache, or nausea at the time of PIIRS diagnosis, whereas the number of patients with a change in hearing increased. Additionally, CSF opening pressure, WBC count, and CSF/serum glucose ratio remained stable, whereas protein levels increased.

### Corticosteroid Therapy for Post-Infectious Inflammatory Response Syndrome

Five patients (38.5%) were treated with pulse corticosteroid therapy of 1000 mg of methylprednisolone for 5–7 days, while 8 patients (61.5%) received corticosteroid regimens of approximately 1 mg/kg daily of prednisone equivalent. Among those receiving pulse corticosteroid therapy, all patients subsequently received oral corticosteroids at a prednisone-equivalents dose of 1 mg/kg/day (IQR, 0.83–1.50 mg/kg/day) for an initial period of 30 days (IQR, 13–35 days), followed by gradual tapering according to their clinical response. The median duration of corticosteroid therapy was 24 weeks (IQR, 7–44 weeks). Among those receiving non-pulse regimens, 5 patients (38.5%) received prednisone, 2 patients (15.4%) received dexamethasone, and 1 patient (7.7%) received methylprednisolone. The corticosteroid dosage for the non-pulse patients, converted to prednisone equivalents, was 0.92 mg/kg/day (IQR, 0.48–1.92 mg/kg/day). The initial dosage was maintained for 14 days (IQR, 8–28 days), and the median duration of corticosteroid therapy was 21 weeks (IQR, 5–27 weeks). All patients received prophylaxis against *Pneumocystis jirovecii* pneumonia, 10 receiving sulfamethoxazole-trimethoprim and 3 atovaquone.

### Clinical Outcomes

**Figure 1.**
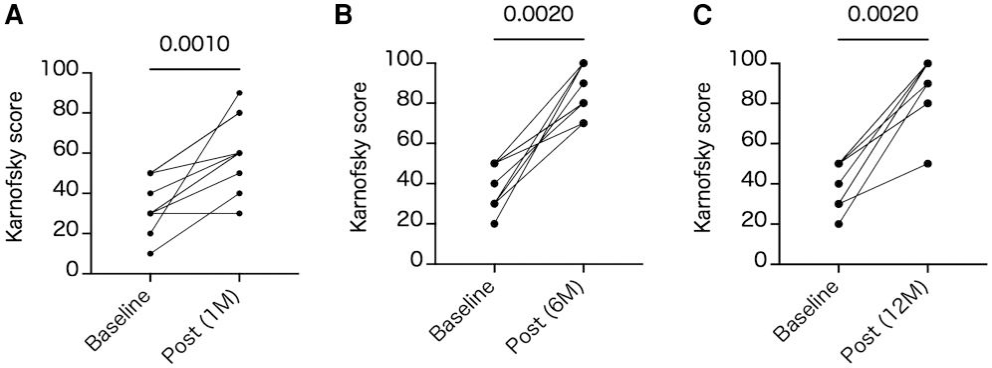
Corticosteroid therapy improves Karnofsky performance scores in patients with cryptococcal PIIRS. Karnofsky scores for the indicated number of patients were obtained at baseline and at *A*, 1 m (n = 12), *B*, 6 m (n = 10), and *C*, 12 m (n = 10) following initiation of corticosteroid therapy. Abbreviations: PIIRS, post-infectious inflammatory response syndrome.

**Figure 2.**
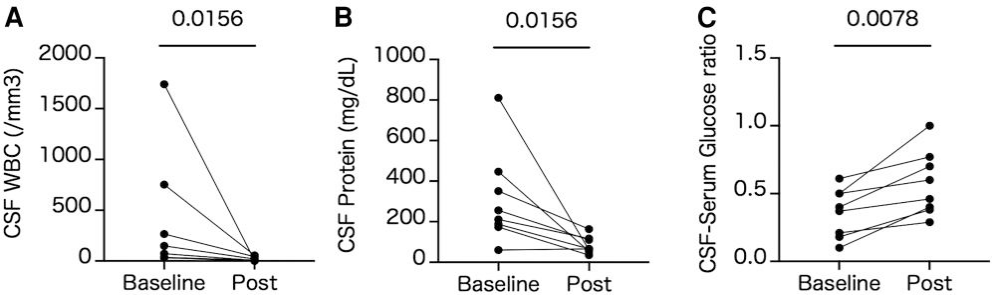
Improvements in CSF parameters are noted at 1 m following corticosteroid therapy. *A*, CSF WBC (n = 7), *B*, protein (n = 8), and *C,* CSF/serum glucose ratio (n = 8). Abbreviations: CSF, cerebrospinal fluid; WBC, white blood cells.

**Figure 3.**
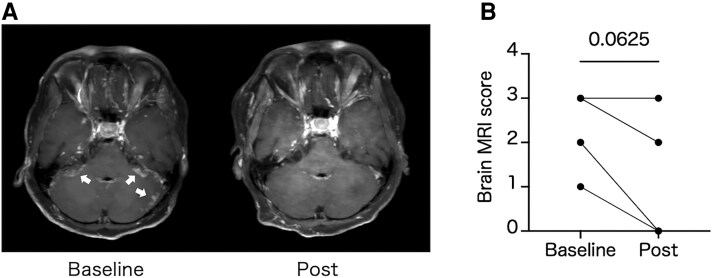
Improvements in MRI inflammatory findings are noted at 1 m following corticosteroid therapy. *A*, Representative post-contrast FLAIR image at the time of PIIRS diagnosis and 1 m after corticosteroid therapy. White arrows indicate cerebellar leptomeningeal enhancement. *B*, Brain MRI score (n = 6). Abbreviations: MRI, magnetic resonance imaging; PIIRS, post-infectious inflammatory response syndrome.

Compared to baseline, Karnofsky scores significantly improved 1 month after corticosteroid therapy in 12 of 13 (*P* = .001) patients at the time of PIIRS diagnosis (Figure 1*A*). At 6-month and 1-year follow-up from PIIRS diagnosis, clinical data was available for all surviving patients. All 10 surviving patients maintained a favorable response with a normal Glasgow coma score of 15 and persistently improved Karnofsky scores at both 6 months and 1 year (*P* = .002; Figure 1*B, C*) as well as self-reported vision and hearing deficits resolving. Two of 3 patients demonstrated resolved reported vision changes at 1 month and 2 of 4 patients showed resolution of hearing deficits. Additionally, significant improvements were observed in CSF parameters at 1 month of those having lumber punctures, including WBC count (*P* = .016; Figure 2*A*), protein levels (*P* = .016; Figure 2*B*), and CSF/serum glucose ratio (*P* = .008; Figure 2*C*). Among the 5 patients with elevated opening pressure, follow-up opening pressures at 1 month after corticosteroid therapy were measured in 3 patients. The median CSF opening pressure decreased from 32 cmH_2_O (IQR, 28.5–34.5 cmH_2_O) to 25 cmH_2_O (IQR, 13–33 cmH_2_O). Brain MRI imaging showed improvements in 4 of 6 patients having repeat imaging at 1 month with resolution of the most common finding of leptomeningeal enhancement with the remainder demonstrated either stable pachymeningitis or leptomeningitis (Figure 3*A*, *B*). Among the 4 patients with hydrocephalus, 2 underwent ventriculoperitoneal shunt placement, 1 received a lumbar drain, and 1 patient showed spontaneous resolution of hydrocephalus without any surgical intervention. CSF cytokine data were available in 2 patients. In 1 patient, IL-6 decreased from 1160 to 46 pg/mL 2 weeks after pulse corticosteroid therapy, while in another, IL-6 and soluble CD25 decreased from 9.8 to 3.5 pg/mL and from 1398.4 to 48.4 pg/mL, respectively, 1 week after starting 1 mg/kg/day corticosteroid therapy. Although limited, these findings paralleled clinical improvement.

Three patients (23.1%) died within 1 year after the diagnosis of PIIRS. The first patient, with severe CNS vasculitis and profound debility, was receiving daily corticosteroids at approximately 1 mg/kg/day and died on day 22 due to septic shock attributable to *methicillin-resistant Staphylococcus aureus* pneumonia. The second patient, with decompensated cirrhosis, received pulse corticosteroid therapy followed by oral corticosteroids at 1 mg/kg/day and died on day 77 due to multifactorial causes, including complications of alcoholic cirrhosis. The third patient, with systemic lupus erythematosus on multiple immunosuppressive agents, was receiving daily corticosteroids at approximately 1 mg/kg/day and died on day 124 due to liver failure in the setting of systemic lupus erythematosus with possible complications from steroid-induced myopathy. The Karnofsky performance scores at PIIRS diagnosis were 10, 30, and 10, respectively, reflecting poor baseline functional status. Two patients (15.4%) experienced weakness of the extremities, and 2 patients (15.4%) had clinical relapse during corticosteroid tapering (Table 3). Among the latter 2 patients, 1 was initiated on corticosteroid therapy at 0.5 mg/kg/day while continuing tacrolimus and the other was started on corticosteroids at 2.0 mg/kg/day with improvement in both; however, symptoms relapsed after discontinuation of corticosteroids at 6 weeks in the second requiring a second short course of oral corticosteroids followed by resumption of the taper.

**Table 3.** Adverse Outcomes in patients with post-infectious inflammatory response syndrome (PIIRS)

Variables	N = 13
Death	3 (23.1)
Cause of death	…
Septic shock^a^	1 (7.7)
Liver failure^b^	1 (7.7)
Other^c^	1 (7.7)
Sequelae	…
Weakness of the extremities	2 (15.4)
Relapse	2 (15.4)

Abbreviation: PIIRS, post-infectious inflammatory response syndrome.

^a^The patient died on d 22.

^b^The patient died on d 124.

^c^The patient died on d 77 due to multifactorial causes, including complications of alcoholic cirrhosis.

## DISCUSSION

The present study demonstrates the effectiveness of corticosteroid regimens for PIIRS in immunocompromised individuals, including solid organ transplant recipients, thereby extending previous findings in apparently healthy individuals [16] to the non-HIV, immunosuppressed population who show poor clinical responses to standard antifungal therapy. First, it is important to distinguish this study from that by Beardsley et al. who found an adverse outcome after corticosteroid use in HIV-associated cryptococcal meningitis. In that study, the HIV-infected population was profoundly immunocompromised with low CD4 counts at the time of corticosteroid initiation and was at risk of increased mortality from opportunistic infections after additional corticosteroid immunosuppression [20]. In contrast, poor clinical responses in non-HIV populations with cryptococcal meningitis such as transplant recipients and the previously healthy have been ascribed to inflammatory reconstitution syndromes [21] or paradoxical inflammatory syndromes [8], respectively. In the previously healthy population, extensive demonstration of elevated CSF inflammatory parameters was thought to result from released antigens after fungal lysis from amphotericin B and flucytosine therapy. In the present population, patients had their immunosuppression reduced after diagnosis, which could have played a factor in reconstituting the immune system, combining with that induced by liberated protein and carbohydrate antigens after fungal lysis by amphotericin B and flucytosine [8]. While the patients in the present study could be considered to have a mixed IRIS/PIIRS syndrome, we have chosen to use the term “PIIRS” in non-HIV associated cases, understanding the heterogeneity of the inflammatory syndrome. Elevated CSF WBC counts and post-contrast leptomeningeal enhancement, choroiditis and ependymitis, similar to PIIRS in the previously healthy population [22], further supported an inflammatory etiology for the poor clinical progression in the present study. It is important to note that patients in our study were on low doses of prednisone dosages at the time of onset of the inflammatory syndrome, which apparently failed to control inflammation-related symptoms.

In the present study, 10 of 13 patients demonstrated prolonged and durable improvement in mental and functional status in this high-risk patient population. This is in contrast to a non-corticosteroid-treated, mixed population of patients with non-HIV who had poor initial clinical responses measured by Montreal Cognitive Assessment scores less than 22 of which only 1 of 8 recovered normal scores over the succeeding 1-year interval [23]. Patients in the present study responded to either high dose pulse (methylprednisolone 1000 mg IV daily × 5–7 days) or more modest doses (equivalent to prednisone 1 mg/kg daily) but required a taper over a median of 21–24 weeks, similar to that in the previously healthy cohort described earlier [24]. Despite this, 2 patients treated with corticosteroids experienced immunological flairs evidenced by clinical deterioration despite negative CSF cultures and/or stable serum cryptococcal antigens, along with worsening CSF findings or recurrence of lesions in previously inflamed areas on brain MRI. Both responded with a short oral increase in prednisone dosage followed by a return to the taper regimen. Importantly, the fact that fungal cultures in all patients remained negative following the initiation of therapy stresses the safety from fungal recurrence when using corticosteroids, similar to that recorded in the previously healthy population. This is undoubtedly due to the continuation of oral fluconazole throughout corticosteroid therapy. Three deaths were recorded in the present study, somewhat expected based on the presence of multiple comorbidities in this high-risk population. Of these, 2 patients showed stabilization or improvement in brain MRI findings consistent with PIIRS following corticosteroid therapy; however, 1 died due to multifactorial causes associated with alcoholic cirrhosis, and another due to liver failure in the setting of systemic lupus erythematosus, with possible contributions from steroid-related myopathy, a known complication of corticosteroids [25, 26]. A third patient died because of sepsis that was likely at least partially a consequence of additional corticosteroid immunosuppression [27]. Such an event underscores the potential risks of corticosteroid therapy and highlights the need for close follow-up in these highly immunosuppressed patients. However, the observed mortality of 23% in this cohort was lower than the 25–42% reported among HIV-negative patients with cryptococcal meningitis [28–30]. This observation suggests a potential benefit for treatment of PIIRS in selected patients and indicates that careful monitoring and management, including concomitant antifungal therapy, may mitigate the risks associated with corticosteroid therapy. Indeed, at the NIH clinical center, we have instituted and now recommend a mitigation strategy of screening blood cultures twice a week in hospitalized patients undergoing combination corticosteroid/immunosuppressive therapy. This strategy successfully detected and enabled early treatment of an asymptomatic bacteremia due to pneumatosis coli in a patient with PIIRS receiving corticosteroid therapy plus additional ruxolitinib at the NIH.

Limitations of this study include the small sample size, incomplete data collection, and the lack of randomization. The study population comprised patients who experienced clinical deterioration despite standard antifungal therapy and were referred for consultation, making it ethically challenging to assign them to a placebo group. Additionally, not all patients had complete longitudinal clinical, CSF, and imaging data, which may have introduced bias. Nevertheless, this represents the largest study to date describing corticosteroid therapy outcomes in non-HIV, immunosuppressed patients with PIIRS. It is also possible that some patients exhibited features of both IRIS and PIIRS. However, distinguishing between these syndromes in immunosuppressed individuals is often challenging in clinical practice. Notably, patients in the present study responded to treatment of the inflammatory syndrome regardless of the degree of immune reconstitution.

In summary, the present study suggests that corticosteroids may provide clinical benefit in immunosuppressed or transplant patients as salvage therapy with PIIRS who experience clinical deterioration despite effective antifungal therapy. Although randomized controlled trials would provide the highest level of evidence, conducting such trials in this rare and high-risk population is likely to be challenging. As a practical next step, the establishment of a national registry could facilitate the systematic collection of clinical data on corticosteroid use, thereby helping to evaluate its safety and efficacy and informing the design of future trials.

## Supplementary Material

jiaf620_Supplementary_Data

## Supplementary Data

Supplementary materials are available at The Journal of Infectious Diseases online (http://jid.oxfordjournals.org/). Supplementary materials consist of data provided by the author that are published to benefit the reader. The posted materials are not copyedited. The contents of all supplementary data are the sole responsibility of the authors. Questions or messages regarding errors should be addressed to the author.

## Notes

***Author Contributions***. Conception and design: Y. I. T. A. H., S. A., and P. R. W., Acquisition and analysis of data: Y. I., T. A. H., L. R., J. S. L., M. K., J. M. S., A. F., L. W. W., R. K. A., S. S., O.E.B., S. C. O., C. N. V., I. S., A. H., and P. R. W., Interpretation of data: Y. I., S. A., and P. R. W., Drafting of the manuscript: Y.I. and P.R.W., Critical revision for intellectual content: S. A., J. S. L., J. M. S., S. C. O., A. H., and P. R. W., Final approval of the version to be published: All authors, Accountability for all aspects of the work: All authors.

***Financial support.*** This research was supported in part by the Division of Intramural Research of the National Institute of Allergy and Infectious Diseases (NIAID), National Institutes of Health (NIH) (AI001123 and AI001124 to P. R. W).

***Data Availability***. The data that support the findings of this study are available from the corresponding author upon reasonable request.

***Potential conflicts of interest.*** J. M. S. reports receiving grants from the National Institute of Allergy and Infectious Diseases of the National Institutes of Health (K23-AI187748) and the Duke University Office for Translation and Commercialization; receiving royalties from Biomeme for patents for gene expression classifiers of fungal infection and from McGraw Hill Publishing; receiving honoraria for a lecture at the University of Michigan ID Grand Rounds; receiving travel support from the University of Michigan ID Grand Rounds, the Mycoses study Group and Education Consortium, and the American Society of Transplantation; and holding patents for gene expression classifiers of fungal infection. R. K. A. reports receiving study/grant support from Aicuris, AstraZeneca, Merck, Takeda, and United Therapeutics. S. S. reports receiving grants from Ansun, Basilea, F2G, and Zeteo Tech Inc.; receiving consulting fees from F2G and Karius; receiving honoraria for lectures from Avir; serving on data monitoring boards for Adamis, Cidara, Pfizer, Basilea, Moderna, and Scynexis; and serving on the Board of Governors of the American College of Physicians. S. C. O. reports receiving honoraria for lectures from Asahikasei and Amgen. C. N. V. D. reports receiving grants or research funding from ACTG (UM1 A1069423), Merck, ViiV Healthcare, and Gilead; receiving honoraria for serving on speaker bureaus for ViiV Healthcare, Gilead, and Merck; and receiving support for travel and accommodations for speaker training from ViiV Healthcare and Gilead. All other authors declare no conflicts of interest.

## References

[1] Pyrgos V, Seitz AE, Steiner CA, Prevots DR, Williamson PR. Epidemiology of cryptococcal meningitis in the US: 1997–2009. PLoS One 2013; 8:e56269. 23457543 10.1371/journal.pone.0056269 PMC3574138

[2] Castelblanco RL, Lee M, Hasbun R. Epidemiology of bacterial meningitis in the USA from 1997 to 2010: a population-based observational study. Lancet Infect Dis 2014; 14:813–9. 25104307 10.1016/S1473-3099(14)70805-9

[3] Pappas PG . Cryptococcal infections in non-HIV-infected patients. Trans Am Clin Climatol Assoc 2013; 124:61–79. 23874010 PMC3715903

[4] Benedict K, Smith DJ, Gold JAW. Epidemiology of Cryptococcosis among patients with commercial health insurance and patients with medicaid, United States, 2016–2022. Open Forum Infect Dis 2024; 11:ofae260. 38798897 10.1093/ofid/ofae260 PMC11127481

[5] Brizendine KD, Baddley JW, Pappas PG. Predictors of mortality and differences in clinical features among patients with Cryptococcosis according to immune status. PLoS One 2013; 8:e60431. 23555970 10.1371/journal.pone.0060431 PMC3608592

[6] Pirofski LA, Casadevall A. Immune-mediated damage completes the parabola: Cryptococcus neoformans pathogenesis can reflect the outcome of a weak or strong immune response. mBio 2017; 8:e02063-17. 10.1128/mBio.02063-17 PMC5727418 29233901

[7] Mook-Kanamori BB, Geldhoff M, van der Poll T, van de Beek D. Pathogenesis and pathophysiology of pneumococcal meningitis. Clin Microbiol Rev 2011; 24:557–91. 21734248 10.1128/CMR.00008-11 PMC3131058

[8] Panackal AA, Wuest SC, Lin YC, et al Paradoxical immune responses in non-HIV cryptococcal meningitis. PLoS Pathog 2015; 11:e1004884. 26020932 10.1371/journal.ppat.1004884 PMC4447450

[9] Williamson PR . Post-infectious inflammatory response syndrome (PIIRS): dissociation of T-cell-macrophage signaling in previously healthy individuals with cryptococcal fungal meningoencephalitis. Macrophage (Houst) 2015; 2:e1078. 27064474 10.14800/Macrophage.1078 PMC4825797

[10] Seher Anjum PW . Clinical aspects of immune damage in cryptococcosis. Curr Fungal Infect Rep 2019; 13:99–108. 33101578 10.1007/s12281-019-00345-7 PMC7580832

[11] Decote-Ricardo D, LaRocque-de-Freitas IF, Rocha JDB, et al Immunomodulatory role of capsular polysaccharides constituents of Cryptococcus neoformans. Front Med (Lausanne) 2019; 6:129. 31275938 10.3389/fmed.2019.00129 PMC6593061

[12] Singh N, Lortholary O, Alexander BD, et al An immune reconstitution syndrome-like illness associated with Cryptococcus neoformans infection in organ transplant recipients. Clin Infect Dis 2005; 40:1756–61. 15909263 10.1086/430606

[13] Anjum S, Dean O, Kosa P, et al Outcomes in previously healthy cryptococcal meningoencephalitis patients treated with pulse taper corticosteroids for post-infectious inflammatory syndrome. Clin Infect Dis 2021; 73:e2789–98. 33383587 10.1093/cid/ciaa1901 PMC8563180

[14] Romani L, Williamson PR, Di Cesare S, et al Cryptococcal meningitis and post-infectious inflammatory response syndrome in a patient with X-linked hyper IgM syndrome: a case report and review of the literature. Front Immunol 2021; 12:708837. 34335625 10.3389/fimmu.2021.708837 PMC8320724

[15] Shoham S, Thapaliya S, Avery R, et al Multiple dimensions of neurological injury in a liver transplant recipient with cryptococcal meningitis. ASM Case Reports 2024; 1:e00040-24. 41245141 10.1128/asmcr.00040-24 PMC12530241

[16] Miller C, Daugherty R, McCulloch M, Stephens I, Williamson PR. Immune reconstitution inflammatory syndrome complicating cryptococcal meningitis in a pediatric heart transplant patient. Pediatr Infect Dis J 2022; 41:145–7. 34609105 10.1097/INF.0000000000003335

[17] Donnelly JP, Chen SC, Kauffman CA, et al Revision and update of the consensus definitions of invasive fungal disease from the European organization for research and treatment of cancer and the mycoses study group education and research consortium. Clin Infect Dis 2020; 71:1367–76. 31802125 10.1093/cid/ciz1008 PMC7486838

[18] Services . USDoHaH. Human subjects protections, Subpart A. 45 CFR Part46; §46.102 Definitions Available at: https://www.ecfr.gov/on/2018-07-19/title-45/section-46.102. Accessed 18 June. 2025 2025.

[19] Singh N, Alexander BD, Lortholary O, et al Cryptococcus neoformans in organ transplant recipients: impact of calcineurin-inhibitor agents on mortality. J Infect Dis 2007; 195:756–64. 17262720 10.1086/511438 PMC2746485

[20] Beardsley J, Wolbers M, Kibengo FM, et al Adjunctive dexamethasone in HIV-associated cryptococcal meningitis. N Engl J Med 2016; 374:542–54. doi:10.1056/NEJMoa1509024 26863355 PMC4778268

[21] Sun HY, Singh N. Opportunistic infection-associated immune reconstitution syndrome in transplant recipients. Clin Infect Dis 2011; 53:168–76. 21690625 10.1093/cid/cir276

[22] Hammoud DA, Mahdi E, Panackal AA, et al Choroid plexitis and ependymitis by magnetic resonance imaging are biomarkers of neuronal damage and inflammation in HIV-negative cryptococcal meningoencephalitis. Sci Rep 2017; 7:9184. 28835663 10.1038/s41598-017-09694-0 PMC5569007

[23] Marr KA, Sun Y, Spec A, et al A multicenter, longitudinal cohort study of cryptococcosis in human immunodeficiency virus-negative people in the United States. Clin Infect Dis 2020; 70:252–61. 30855688 10.1093/cid/ciz193 PMC6938979

[24] Anjum S, Dean O, Kosa P, et al Outcomes in previously healthy cryptococcal meningoencephalitis patients treated with pulse—taper corticosteroids for post-infectious inflammatory syndrome. Clin Infect Dis 2021; 73:e2789–98. 33383587 10.1093/cid/ciaa1901 PMC8563180

[25] Bessone F, Poles N, Roma MG. Challenge of liver disease in systemic lupus erythematosus: clues for diagnosis and hints for pathogenesis. World J Hepatol 2014; 6:394–409. 25018850 10.4254/wjh.v6.i6.394 PMC4081614

[26] Wu M, Liu C, Sun D. Glucocorticoid-induced myopathy: typology, pathogenesis, diagnosis, and treatment. Horm Metab Res 2024; 56:341–9. 38224966 10.1055/a-2246-2900

[27] Minneci PC, Deans KJ, Eichacker PQ, Natanson C. The effects of steroids during sepsis depend on dose and severity of illness: an updated meta-analysis. Clin Microbiol Infect 2009; 15:308–18. 19416302 10.1111/j.1469-0691.2009.02752.x PMC3383780

[28] Motoa G, Pate A, Chastain D, et al Increased cryptococcal meningitis mortality among HIV negative, non-transplant patients: a single US center cohort study. Ther Adv Infect Dis 2020; 7:2049936120940881. 32685148 10.1177/2049936120940881 PMC7346692

[29] Namie H, Takazono T, Hidaka Y, et al The prognostic factors for cryptococcal meningitis in non-human immunodeficiency virus patients: an observational study using nationwide database. Mycoses 2024; 67:e13658. 37807638 10.1111/myc.13658

[30] Teekaput C, Yasri S, Chaiwarith R. Cryptococcal meningitis: differences between patients with and without HIV-infection. Pathogens 2023; 12:427. 36986349 10.3390/pathogens12030427 PMC10051108

