# Supplementary material for: Corticosteroid Therapy and Long-Term Outcomes of Post-Infectious Inflammatory Syndrome in Non-HIV Immunosuppressed Cryptococcal Meningitis: A Multicenter Case Series
Source: J Infect Dis. 2026 Jan 5;233(2):e553–60. doi: 10.1093/infdis/jiaf620 (PMC12861043; doi:10.1093/infdis/jiaf620)
Supplement: jiaf620_Supplementary_Data [file jiaf620_supplementary_data.docx]

| **Table S1. Collaboration Institutions** |  |
| --- | --- |
|  |  |
| **Institution^*^** | **Location** |
| Bambino Gesù Children’s Hospital | Rome, Itary |
| Brigham and Women's Hospital | Boston, MA, USA |
| Cone Health | Greensboro, NC, USA |
| Dana-Farber Cancer Institute | Boston, MA, USA |
| Duke University School of Medicine | Durham, NC, USA |
| Johns Hopkins University School of Medicine | Baltimore, MD, USA |
| Oregon Health and Science University | Portland, OR, USA |
| UCLA David Geffen School of Medicine | Los Angeles, CA, USA |
| University of Alabama at Birmingham | Birmingham, AL, USA |
| University of Virginia Children`s Hospital | Charlottesville, VA, USA |
| University of Washington School of Medicine | Seattle, WA, USA |
| University of Wisconsin School of Medicine and Public Health | Madison, WI, USA |
| * Institution names are listed in alphabetical order. | |
